# A randomized controlled trial evaluating the impact of selective axillary nerve block after arthroscopic subacromial decompression

**DOI:** 10.1186/s12871-020-0952-y

**Published:** 2020-01-31

**Authors:** Christian Rothe, Jørgen Lund, Morten Troels Jenstrup, Christian Steen-Hansen, Lars Hyldborg Lundstrøm, Asger Mølgaard Andreasen, Kai Henrik Wiborg Lange

**Affiliations:** 1 Department of Anaesthesiology, Nordsjællands Hospital, University of Copenhagen, Dyrehavevej 29, DK-3400 Hillerød, Denmark; 2 Department of Anaesthesiology, Aleris-Hamlet Hospital, Copenhagen, Denmark

**Keywords:** Anatomy, Regional anesthesia, Selective nerveblocks, Axillary nerve

## Abstract

**Background:**

The sensory innervation of the shoulder is complex and there are variations in the branching patterns of the sensory fibres. Articular branches from the axillary nerve to the subacromial bursa are described in more than 50% of investigated shoulders but the isolated contribution of sensory input from the axillary nerve has never been investigated clinically.

We hypothesized that a selective block of the axillary nerve would reduce morphine consumption and pain after arthroscopic subacromial decompression.

**Methods:**

We included 60 patients in a randomized, blinded, placebo-controlled study. Patients were randomized to a preoperative selective ultrasound-guided axillary nerve block with 20 mL ropivacaine (7.5 mg/mL) or 20 mL saline. Primary outcome was intravenous morphine consumption 0–4 h postoperatively. Secondary outcome was postoperative pain evaluated by a visual analogue scale (VAS) score (0–100).

**Results:**

We analysed data from 50 patients and found no significant difference in 0–4 h postoperative morphine consumption between the two groups (ropivacaine 14 mg, placebo 18 mg (*P* = 0.12))*.* There was a reduction in postoperative pain: VAS 0–4 h (area under the curve) (ropivacaine 135, placebo 182 (*P* = 0.03)), VAS after 8 h (ropivacaine 9, placebo 20 (*P* = 0.01)) and VAS after 24 h (ropivacaine 7, placebo 18 (*P* = 0.04)). Eight out of 19 patients with a successful selective axillary nerve block needed an interscalene brachial plexus escape block.

**Conclusions:**

Selective block of the axillary nerve has some pain relieving effect, but in this setting the effect was unpredictable, variable and far from sufficient in a large proportion of the patients.

**Trial registration:**

ClinicalTrials.gov (NCT01463865). Registered: November 1, 2011.

## Background

The most common types of shoulder surgery are hemiarthroplasty, total shoulder arthroplasty, shoulder arthroscopy, subacromial decompression and shoulder stabilizing procedures. Interscalene brachial plexus block (IBPB) block is effective for analgesia in all procedures and for anaesthesia and analgesia in arthroscopic procedures. Very rare but serious complications to the IBPB are often mentioned as the primary concern for using this nerve block [1–5]. An important clinical consideration is the risk of rebound pain when the IBPB wears off. Studies indicate that selective nerve blocks might be less effective the first 8 h postoperatively but hereafter the patients have better pain control [6]. Furthermore, patient comfort after selective nerve blocks is increased because only muscles around the shoulder are blocked and the patient is able to move the forearm [1].

The innervation of the shoulder is complex and involves several nerves. The most important nerves are the axillary nerve and the suprascapular nerve but other nerves also contribute to the sensory innervation [7–9]. A selective suprascapular nerve block can reduce the pain and morphine consumption after arthroscopic shoulder surgery [10]. Studies of a combined axillary nerve and suprascapular nerve (SSN) block have demonstrated similar effects [11–17].

We hypothesized that a selective block of the axillary nerve would reduce morphine consumption after arthroscopic subacromial decompression (ASD). We chose to study patients scheduled for ASD for two reasons. 1) A cadaver study found articular branches from the axillary nerve to the subacromial bursa in more than 50% of the investigated shoulders [18] 2) A case study indicated a positive effect of a selective ultrasound-guided block of the axillary nerve (SBAN) on postoperative pain after ASD [19].

## Methods

### Patients and design

This single center, randomized, blinded, placebo-controlled study was performed in compliance with the declaration of Helsinki and its amendments and was conducted according to the principles of Good Clinical Practice [20]. The trial was registered at ClinicalTrials.gov (NCT01463865) and approved by the Ethics Committee of the Capital Region of Denmark (H-1-2011-057) and the Danish Data Protection Agency. Our manuscript was reported according to CONSORT (CONsolidated Standards of Reporting Trials) guidelines.

From November 2011 to December 2013, we screened elective patients scheduled for ASD at Aleris-Hamlet Hospital, Copenhagen, Denmark, for inclusion in the study.

**Fig. 1:**
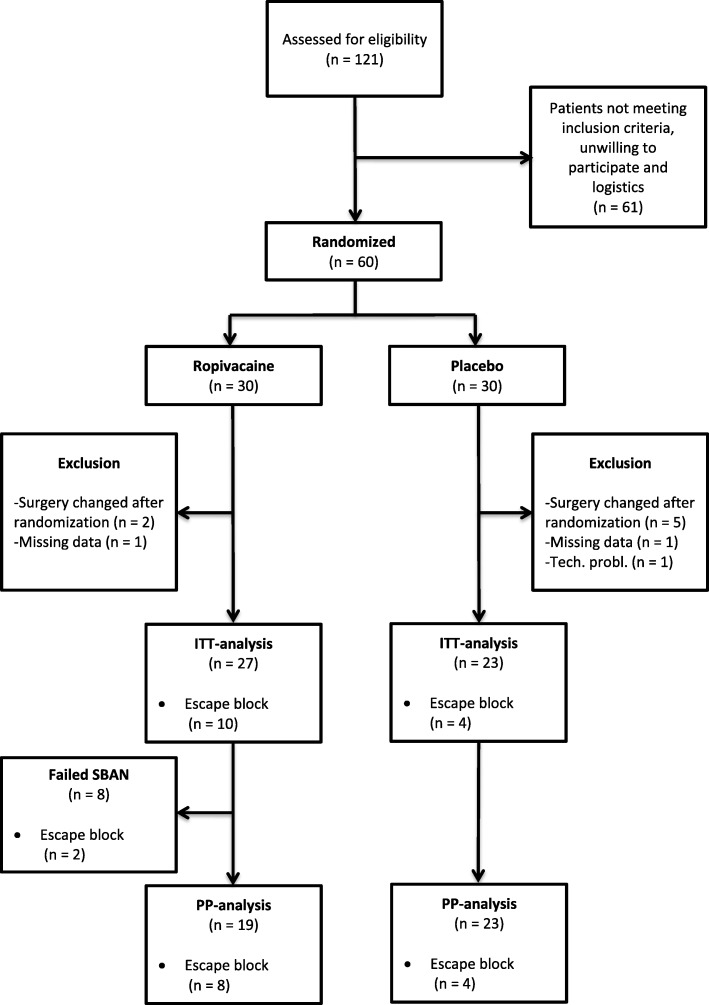
CONSORT Flow diagram of patients enrolled in the study

Exclusion criteria were: age < 18 years, body mass index ≥40 kg.m^− 2^, American Society of Anesthesiologist classification (ASA) IV, preoperative opioid use or allergy to drugs used in the study. Patients scheduled for ASD who had a change of procedure intraoperatively were excluded after inclusion (Fig. 1). We obtained written informed consent from all patients before inclusion.

### Randomisation, allocation concealment and blinding

Based on a central computer-generated list, the patients were randomized in a 1:1 ratio and in blocks of 6 to receive either a preoperative SBAN or placebo. Allocation concealment was ensured according to the principles of SNOSE (Sequentially Numbered Opaque Sealed Envelopes). A health professional with no relation to the study or study location prepared 60 opaque envelopes containing instructions for study medication preparation with either 20 mL isotonic saline or 20 mL ropivacaine, 7.5 mg.ml^− 1^ (B Braun Medical A/S, Frederiksberg, Denmark). A nurse with no relation to the study prepared the medication. To reduce performance bias, the patients, caregivers, the physicians performing the blocks and investigators were blinded to allocation. Further, to reduce detection bias, the physicians assessing the outcome measurements were blinded to allocation. The randomization list was kept in a sealed envelope and separated from the investigators until data collection was completed and the data was validated.

### Interventions

**Fig. 2:**
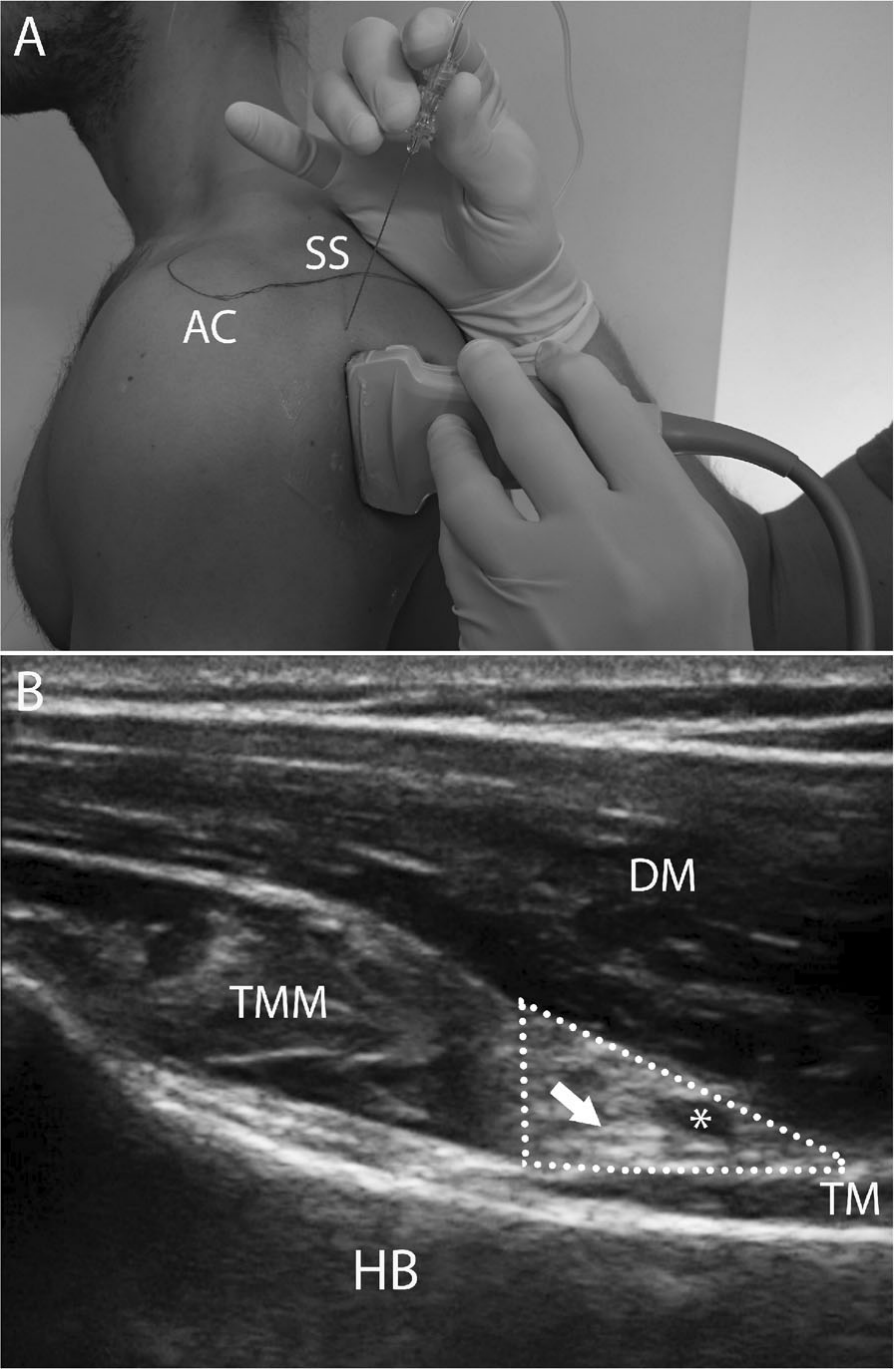
**a** Surface anatomy and transducer position of selective axillary nerve block. The acromion (AC) and the scapular spine (SS) are marked. **b** Ultrasonographic image of the shoulder region with the transducer positioned as in Fig. 1. **a** Important landmarks are the deltoid muscle (DM), the humeral bone (HB), the teres minor muscle (TMM) in transverse section and the triceps muscle (TM) in longitudinal section. The axillary nerve (arrow) is cranial to the posterior circumflex humeral artery (*) in the neurovascular space (dotted triangle)

SBAN: We used a 13–5 MHz linear transducer (GE logiq e and 12 L transducer; GE Healthcare, Milwaukee, WI) to visualize the posterior circumflex humeral artery in the neurovascular space on the posterior aspect of the humeral neck. We chose a transducer position giving the best short axis view of the posterior circumflex humeral artery, where the artery leaves the quadrangular space and winds around the neck of the humerus. The ultrasonographic landmarks were the neck of the humerus, the deltoid muscle, the teres minor muscle, the triceps brachii muscle and the posterior circumflex humeral artery (Fig. 2). We used an 80 mm 22G insulated nerve stimulation needle (B. Braun Melsungen AG, Melsungen, Germany) and injected 20 mL of study medication into the neurovascular plane as described in a previous study [21]. CR, MTJ and JL performed all blocks.

### Assessment of block success

Assessment of block was performed at baseline and 30 min after the nerve block was performed. Sensory function was assessed by cold detection (5 °C cold vial) in the cutaneous area overlying the deltoid muscle and recorded as normal or reduced/absent. Motor function was assessed by maximal voluntary isometric contraction (MVIC) and recorded as normal or reduced/absent deltoid muscle tone. Block success was defined as both reduced/absent sensory and motor function of the axillary nerve. Sensory and motor functions of the musculocutaneous, radial, ulnar and median nerves were tested in a similar way.

### Surgery and anaesthesia

Arthroscopic surgery was performed through two portals with no use of local anaesthetics (LA) either for intra-articular injection or for local infiltration. General anaesthesia was induced with an infusion of remifentanil (0.1–0.3 μg.kg^− 1^.min^− 1^) (Remifentanil “B. Braun”, B. Braun Medical, Frederiksberg, Denmark) followed by a bolus of propofol (2–3 mg.kg^− 1^) (Propofol “B. Braun”, B. Braun Medical, Frederiksberg, Denmark). No volatile agents or neuromuscular blocking agents were used. The patients had a laryngeal airway mask placed and were normoventilated using capnography. Intravenous anaesthesia was maintained with infusions of remifentanil (0.1–0.5 μg.kg^− 1^.min^− 1^) and propofol (6–8 mg.kg^− 1^.h^− 1^). We gave 4 mg of Ondansetron (Zofran®, GlaxoSmithKline Pharma A/S, Brøndby, Denmark) intravenously 15 min before end of surgery and gave 0.5 mg of alfentanil (Rapifen® Janssen-Cilag A/S, Birkerød, Denmark). We moved the patients to the recovery room after surgery and connected a PCA pump set to deliver a 2 mL bolus of morphine 1 mg/mL (SAD Amgros I/S, Copenhagen, Denmark) with a lockout time of 10 min. All patients were instructed to use the PCA pump to reduce their pain to a visual analogue scale (VAS) score (0–100) < 30. The patients could request an extra dose of 5 mg morphine in case the max PCA administration was insufficient up to three times. We performed an ultrasound-guided IBPB escape block with 5 mL ropivacaine 7.5 mg x ml^− 1^ if the VAS score was above 70 despite PCA and max escape morphine within the first four postoperative hours. After 4 h the patients were discharged. All patients were prescribed a daily oral dose of paracetamol 1 g × 4 (Pinex® Actavis A/S, Gentofte, Denmark), ibuprofen 400 mg × 3 (Ibumetin® Takeda Pharma A/S, Taastrup, Denmark) from the day of surgery. For postoperative pain management we added morphine 10 mg × 1 (Morfin “DAK”, Takeda Pharma A/S, Taastrup, Denmark) as needed (maximal daily dose: 50 mg).

**Outcomes.** Primary outcome: Intravenous morphine consumption during the first four postoperative hours (intravenous morphine consumption 0–4 h). Secondary outcomes: 1) Postoperative pain during the first four postoperative hours (VAS 0–4 (AUC)); 2) Postoperative pain after 8 h (VAS 8 h); 3) Postoperative pain after 24 h (VAS 24 h); 4) Accumulated oral morphine consumption during the first 24 postoperative hours; 5) Number of escape blocks during the first four postoperative hours; 6) Vomiting and/or nausea during the first four postoperative hours.

### Assessments of outcomes

#### Primary outcome

The accumulated dose of administered intravenous morphine (in mg) during the first four postoperative hours was registered.

#### Secondary outcomes

VAS 0–4 (AUC): Postoperative pain during the first four postoperative hours was assessed at rest every 30 min by asking the patient to rate the pain from the operated site from 0 to 100 mm on a sliding VAS ruler. Zero corresponded to no pain and 100 to the worst possible pain. The eight pain measurements for each patient were condensed to a single measure as AUC. VAS scores 8 and 24 h postoperatively were obtained in a telephone interview on the following day. The patients had prior to discharge been carefully instructed to register their VAS scores at rest 8 and 24 h postoperatively. The accumulated oral morphine consumption during the first 24 h postoperatively was obtained in the same telephone interview by asking the patient. Prior to discharge the patients were carefully instructed to record their oral morphine consumption during the first 24 h after the operation. Escape blocks during the first four postoperative hours were registered and recorded as yes/no. Vomiting and/or nausea during the first 4 h was registered as yes/no.

### Statistical analysis

We chose a reduction in intravenous morphine consumption of 2.5 mg during the first 4 h postoperatively between the placebo group and the ropivacaine group to be clinically significant. This was based on a study with patients undergoing similar surgery where the placebo group had a mean intravenous morphine consumption of 5 mg during the first four postoperative hours [10]. A reduction of 2.5 mg would therefore equal a 50% reduction in morphine consumption between groups in our study. We assumed a standard deviation of 3.0 mg morphine consumption in both groups. With α = 0.05 and a power of 80% 2 × 23 patients were required using sample size calculation comparing two means: two samples, two sided equality (www.powersamplesize.com). We expected to exclude some patients after randomization due to changes in scheduled surgical procedures after randomization. We therefore included 60 patients for randomization. We performed both intention-to-treat and per-protocol analyses in intravenous morphine consumption 0–4 h. Patients with incomplete or failed SBAN defined as both reduced/absent sensory and motor function of the axillary nerve were removed from the intention-to-treat analysis before performing per-protocol analysis. Patients with escape block were removed from secondary outcome analyses because we did not follow up on VAS score and oral morphine consumption after discharge in these patients. We calculated the AUC by linear extrapolation between each VAS score measured at a fixed interval of 30 min. All continuous/scale data are presented as medians (range) and Mann Whitney u test for non-parametric unpaired statistical tests was used to compare differences between the ropivacaine group and the placebo group. Chi-square test was used to compare dichotomous outcomes. Fractions are presented with corresponding 95% confidence intervals (CI). *P* < 0.05 was considered statistically significant. We used SPSS software package (SPSS Statistics, version 19.0.0, 8 SPSS, Chicago, IL, USA) for statistical analyses.

## Results

We randomized 60 patients. After excluding patients because of change in surgical procedure (*n* = 7), missing data (*n* = 2) and technical problems (*n* = 1) 50 patients were included in our primary intention-to-treat analyses (27 in the ropivacaine group and 23 in the placebo group) (Fig. 1). Patient characteristics are listed in Table 1. In total, 14 patients had an escape block (10 in the ropivacaine group and 4 in the placebo group; (*P* = 0.21)). Of the 10 escape blocks in the **ropivacaine** group 8 were performed in patients with a successful SBAN. The SBAN was successful in 19 of the 27 patients in the ropivacaine group (70%, CI 56–88%). There were no harms or unintended effects reported in any of the groups.

**Table 1:** Patient characteristics

	Ropivacaine	Placebo
Gender, (female/male)	11/16	11/12
Age, yr.	53 (32–74)	53 (35–75)
Height, cm	173 (164–198)	171 (160–200)
Weight, kg	79 (51–108)	79 (53–130)
BMI, kg/m^2^	26 (21–35)	26 (21–33)
Dynamic pain, VAS (baseline)	68 (10–100)	67 (4–100)

Values are medians and (range)

### Intention to treat analysis

There was no significant difference in the accumulated intravenous morphine consumption 0–4 h postoperatively between the two groups (ropivacaine group: 14 mg, placebo group: 18 mg; (*P* = 0.12)). Following standard procedure, all patients should have remained included in our intention to treat analyses of our secondary outcomes. However, we did not follow up on VAS-scores in the patients having an escape block. Thus, because of missing values, we had to exclude all patients with escape block before analysing our secondary outcomes concerning pain assessment. Therefore, our analyses of pain scores were only performed in 36 patients (17 in the ropivacaine group and 19 in the placebo group). All secondary outcomes exploring the pain-relieving effect at rest showed significant differences. VAS 0–4 h (AUC) (ropivacaine group: 135, placebo group: 182; (*P* = 0.03)), VAS 8 h (ropivacaine group: 9, placebo group: 20; (*P* = 0.01)) and VAS 24 h (ropivacaine group: 7, placebo group: 18; (*P* = 0.04)). We found no significant difference in oral morphine consumption (ropivacaine group: 0 mg, placebo group: 10 mg; (*P* = 0.25)). Nausea and vomiting were similar in the two groups (ropivacaine group: 2/16 patients, placebo group: 4/18 patients; (*P* = 0.66)) and sensory and motor test in the radial, ulnar and median nerves were not affected after the SBAN in any of the patients. Results are listed in Table 2.

**Table 2:** Outcomes after selective axillary nerve block

	Ropivacaine	Placebo	*P*
*Primary outcomes*			
Intravenous morphine 0–4 h, mg ITT	14 (0–35)	18 (2–34)	0.12
Intravenous morphine 0–4 h, mg PPA^a^	16 (0–35)	18 (2–34)	0.40
*Secondary outcomes*			
Pain (rest) 0–4 h, VAS measured as AUC^b^	135 (4–293)	182 (15–383)	0.030^*^
Pain (rest) 8 h, VAS^b^	9 (0–26)	20 (0–67)	0.01^*^
Pain (rest) 24 h, VAS^b^	7 (0–25)	18 (0–45)	0.04^*^
Oral morphine 4–24 h, mg^b^	0 (0–20)	10 (0–30)	0.25
Escape block (number of patients)	10 of 27 (37%)	4 of 23 (17%)	0.21
Nausea and vomiting 0–4 h^c^	2 of 16 (13%)	4 of 18 (22%)	0.66

Values are medians and (range). *ITT* intention to treat analysis, *PPA* per protocol analysis, *VAS* visual analogue scale and *AUC* area under the curve

^*^P < 0.05

^a^Patients with unsuccessful nerve block removed from analysis (8 ropivacaine, 0 placebo)

^b^Patients with escape block was removed from analysis (10 ropivacaine, 4 placebo group)

^d^Two patients in the ropivacaine group had missing data

### Per protocol analysis

After excluding the 8 patients with failed SBAN from the intention-to-treat analysis our per-protocol analysis of the primary outcome included 42 patients (19 from the ropivacaine group and 23 from the placebo group). There was no difference in the accumulated intravenous morphine consumption 0–4 h postoperatively between the two groups (*P* = 0.40).

### Sensitivity analysis

Because of an unexpected large number of escape blocks, numerous patients were excluded from our assessments of secondary outcomes evaluating pain perception. Therefore, we performed a post hock sensitivity analysis where we assumed that the VAS-score of the patients having an escape block would have persisting severe pain if no escape block was performed. The threshold for performing an escape block was a persistent VAS score above 70. Thus, we chose to impute a VAS score of 85 for all patients with missing VAS-values after having an escape block. Re-analysis with the imputed VAS-values as described included 27 patients in the ropivacaine group and 23 patients in the saline group (Fig. 1) and showed no significant difference in VAS 0–4 h (AUC) (ropivacaine group: 197 (4–614); placebo group: 190 (15–596); (*P* = 0.97)).

No other severe events or uninteded effects were recorded.

## Discussion

To our knowledge, this is the first clinical study evaluating the morphine reducing effect of a SBAN after ASD. We found no difference in intravenous morphine consumption between the ropivacaine group and the placebo group 0–4 h postoperatively. We found a small, but statistically significant, reduction in pain scores during the first 24 h in the ropivacaine group compared to the placebo group. One should always be careful when reporting an essentially negative result. This is also the case in the present study. There are several major weaknesses that deserve further scrutiny.

First of all, one may argue that the primary end-point was not wisely chosen. If we consider SBAN to last an average of 10–12 h, then it would make more sense to measure the primary end point for 10–12 h. This would also diminish the potential blurring (confounding) effect of recovery from propofol sedation which may result in equal morphine consumption between groups during the first 4 h postoperatively.

We also had an unexpected high number of patients with severe postoperative pain despite intravenous morphine administration. These patients – 10 out of 27 in the ropivacaine group and 4 out of 23 in the saline group - had an interscalene brachial plexus escape block. This is a critical limitation. A substantial number of patients were excluded from secondary outcome analyses because we did not follow up on the patients receiving escape blocks. Therefore, the study may have been underpowered for the secondary outcome measures. Because this group represented the patients with the highest pain scores our pain analyses were compromised and therefore less valid. After escape block the morphine consumption was reduced and this could have compromised our primary outcome measure to some extent. We chose to perform a *post* hock sensitivity analysis on VAS-score 0–4 h postoperatively, including the patients from our intention-to-treat analysis who had an escape block. The missing VAS-scores were imputed with a value of 85. Analysing data in this way showed no difference in VAS-scores between the two groups at any time point 0–4 h postoperatively. Obviously, this sensitivity analysis is based on an assumption of constant pain during the remainder of the observation period, which may not be the case. Retrospectively, it seems clear that the escape blocks should only have been administered after the first four postoperative hours. However, we did not forecast the extended need of escape blocks. Further, we considered it ethically more correct to offer a block immediately if bolus morphine could not provide sufficient pain relief.

Actually, the high number of needed escape blocks helps us in the interpretation of the efficacy of the SBAN in the present setting. Eight patients with successful SBAN needed an escape block. The SBAN was therefore far from sufficient for pain relief in a large proportion of the patients (eight out of 19 patients with successful SBAN). It is impossible to know if a higher success rate would have changed the conclusions in favour of a larger pain relieving effect of the SBAN.

We had a high number of failed blocks and there are several explanations for this. The steep needle angle, deep nerve localization and limited possibilities for a parallel shift of the transducer make the SBAN a challenging nerve block. The position of the needle tip must be precise to ensure injection into the neurovascular space. Prior to the present study we performed a case series study to test if the surgical procedure was relevant [19]. We also performed magnetic resonance imaging scans of different volumes of LA to ensure the best spread of LA in the quadrangular space. Twenty ml of LA gave the best visual spread on magnetic resonance imaging but with the experience gained in the present study, the higher volume did not increase the success rate of the block. A smaller volume would probably have been sufficient and safer.

Block success was defined as a combination of sensory and motor block of the axillary nerve. Motor block assessment was performed by palpating the degree of tension in the deltoid muscle during MVIC. It would have been better to actually measure MVIC and predefine a % decrease in MVIC for a successful motor block. However, while it is easy to define the criteria for a successful block, the fulfilment of these criteria does not always translate meaningfully into a clinical context. We might have overestimated the block failure rate because our test of block success partly depended on a simplified manual muscle test. The sensitivity of a manual muscle test is low especially if the muscle force is only decreased by up to 25% [22]. Furthermore, the baseline pain from the shoulder is likely to increase the uncertainty of the test.

It is impossible to know if a higher success rate would have changed our conclusions. However, after performing additional analyses on the data as described above and taking the high number of needed escape blocks into account, we conclude: In this setting, the SBAN has some pain relieving effect, but this effect is unpredictable, variable and far from sufficient in a large proportion of the patients.

The shoulder, like the knee, is a region with complex innervation by multiple nerves. It is likely that the clinical effect of a selective nerve block will vary with different surgical interventions. We therefore recommend uniform surgical procedure in future selective nerve blocks studies for shoulder surgery. Further, studies should be designed to maximize the pain signal and reduce noise. This can be accomplished by measuring pain for a longer time period, i.e. the anticipated mean block duration time and by performing the block postoperatively and only include patients with moderate to severe pain. It is also of value to perform both *intention to treat* and *per protocol analyses* to see how the block performs.

## Conclusion

Selective block of the axillary nerve has some pain relieving effect, but in the present setting this effect was unpredictable, variable and far from sufficient in a large proportion of the patients.

## Data Availability

The protocol and datasets used and/or analysed during the current study are available from the corresponding author on reasonable request.

## Abbreviation

ASA: American Society of Anaesthesiologist classification
ASD: Arthroscopic subacromial decompression
AUC: Area under the curve
CI: 95% confidence intervals
IBPB: Interscalene brachial plexus block
LA: Local anaesthetics
SBAN: Selective ultrasound-guided block of the axillary nerve
SSN: Suprascapular nerve
VAS: Visual analogue scale
